# Expert opinion on a safe same day discharge strategy as standard of care after leadless pacemaker implantation

**DOI:** 10.1016/j.ijcha.2025.101649

**Published:** 2025-03-18

**Authors:** Riyaz Somani, James Daniels, Alexis Mechulan, Vincent Paul, David Sharman, Shirley Sze, Xavier Viñolas Prat

**Affiliations:** a Glenfield Hospital, University Hospitals of Leicester NHS Trust, UK; b Department of Cardiovascular Sciences, University of Leicester, Leicester, United Kingdom; c University of Texas, Southwestern Medical Center, Texas, USA; d Hôpital Privé Clairval, Marseille, France; e Fiona Stanley Hospital, Murdoch, Australia; f Northampton General Hospital NHS Trust, Northampton, UK; g NIHR Leicester Biomedical Research Centre, University of Leicester, UK; h Development Centre for Population Health, University of Leicester, UK; i Hospital Sant Pau, Barcelona, Spain

**Keywords:** Same day discharge, Micra™, Patient pathway

## Abstract

**Introduction:**

Leadless pacemaker (LPs) is a safe and effective alternative to conventional transvenous pacing. There is currently no guidance on which patients could be safely discharged the same day post-procedure.

**Purpose:**

To provide guidance to medical teams regarding safe same day discharge (SDD) after LP implantation.

**Methods:**

An Advisory Board (AB) of 6 expert Micra™ implanters was formed. Interviews were conducted with each member to understand their experience on patient selection, care pathway, complications, and follow-up of Micra™ implanted patients. This information was used to develop a patient pathway for safe SDD after Micra™ implantation. A further survey was conducted to obtain consensus regarding decision points within the pathway.

**Results:**

The SDD after Micra™ Implantation Patient Pathway consists of four phases:

Pre-procedure assessment: Social factors are key in deciding suitability of SDD (6/6 AB members agreed, 100%). Patient’s comorbidities, frailty status and timing of procedure are also important in decision-making for SDD.

Micra™ implant: Ultrasound-guidance reduces vascular access-related complications, increasing the likelihood for SDD (100%).

Post-procedure observation: Peri-procedural complications such as pericardial effusion, severe vascular complications, bleeding from access site and device complications would prevent SDD (100%). Patients should complete 6 h of observation prior to discharge (100%).

Follow-up: First follow-up should be in-person, 1–4 weeks post-procedure (84 %). Long-term follow-up should be organised as per Micra™ standard of care at each centre (100 %).

**Conclusions:**

SDD after Micra™ Implantation Patient Pathway was developed via expert consensus. Adoption of the pathway in clinical practice may facilitate safe SDD after Micra™ Implantation.

## Graphical abstract

Key factors to consider before and after Micra™ implantation when deciding on same day discharge.

**Figure:**
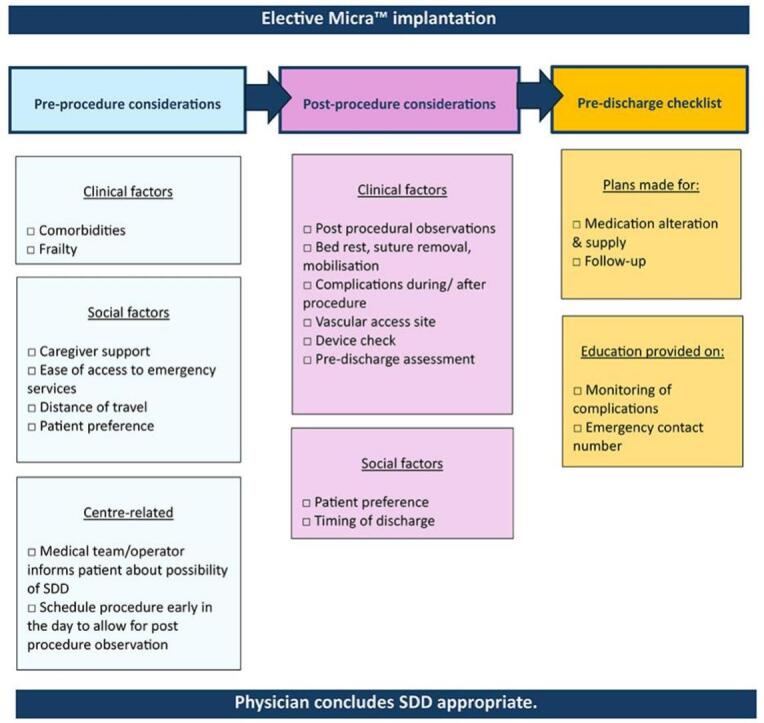

## 1 Introduction

Transvenous pacemakers have long been established as the gold standard treatment for bradyarrhythmia. However, transvenous pacing are still associated with significant complications, including bleeding, infection, pneumothorax, lead displacement or malfunction and pericardial effusion/ cardiac perforation.[1] Leadless pacemakers (LPs) were developed with the intention to mitigate some of the complications of transvenous pacemakers, in particular pocket and lead related complications.

The Micra™ (Medtronic) is a miniaturised LP, first implanted in 2013 during the investigational trial.[2] Up to date, over 200,000 devices have been implanted worldwide.[3] Micra™ is inserted percutaneously through the femoral vein and implanted directly on the ventricular wall using a customised catheter-based delivery system. Although no randomised controlled data comparing outcomes between leadless versus transvenous pacemakers is available, there is extensive evidence regarding the safety and efficacy of the Micra^TM^. [[2], [4], [5]] The European Society of Cardiology pacing guidelines recommend LPs to be considered as an alternative to transvenous pacemakers in patients with no upper extremity venous access and in those with a particularly high risk of device pocket infection, e.g. patients on haemodialysis and those with previous infection (class IIa, level B).[6].

The Heart Rhythm Society/ European Heart Rhythm Association (HRS/ EHRA) guidelines do not advise mandatory overnight hospital stay post cardiovascular implantable electronic devices (CIED). [7] Same day discharge (SDD) post CIED has been carried out mostly in the United States or United Kingdom; there is little data on its implementation in the rest of Europe. [8,9] There is currently a lack of evidence on how to select patients fit for SDD and what is the optimal timing of discharge after Micra™ implantation. The low complication rate and high implant success rate of Micra™ makes SDD a potentially attractive strategy to reduce hospital stay and medical cost. It could also be beneficial for patients as many patients prefer to be discharged as soon as possible. Nevertheless, SDD could only be deemed appropriate when safety concerns are addressed.

This consensus document aims to provide guidance about key clinical, social, and centre-related factors that should be considered before and after the procedure in order to facilitate safe SDD after Micra™ implantation.

## 2 Methods

An Advisory Board (AB) of six expert Micra™ implanters was set up to develop a pathway for the implementation of a safe SDD strategy after Micra™ implantation. Five countries were represented in the AB, including Australia, France, Spain, United Kingdom, and the United States of America. Each expert implanter had performed over 100 implants individually.

The development of the SDD after Micra™ Implantation Patient Pathway involved 4 steps.

• Step 1: Literature review

A review of currently available guidelines and literature on SDD after cardiac implantable electronic device (CIED) implantation, with particular focus on transvenous pacemakers and the Micra™ device, was performed. Medline, Embase and PubMed databases were searched on October 26, 2022, using the following search strategy: ('ambulatory surgery' OR 'same day' OR ‘outpatient’) AND ('cardiac rhythm management device' OR 'leadless pacemaker' OR 'pacemaker implantation' OR 'implanted heart pacemaker' OR ‘pacemaker’) AND (‘transvenous’ OR ‘Micra’ OR ‘medtronic’ OR 'transvenous pacemaker electrode'). A total of 22 publications were selected and reviewed. This information was used to draft a topic guide to conduct interviews with members of the AB to identify key considerations for the implementation of SDD strategy after Micra™ implantation.

• Step 2: Interviews with expert implanters

Individual interviews were conducted with 6 expert implanters to capture their experience on patient selection, existing care pathway, complications, and follow-up of Micra™ implanted patients. A set of questions was included in the interviews to evaluate how various factors could influence the decision for SDD. An initial draft of the SDD after Micra™ Implantation Patient Pathway was formulated based on interview responses. The pathway was structured based on the following four phases: 1) pre-procedure assessment; 2) Micra™ implant; 3) post-procedure observation and discharge and 4) follow-up. Specific questions were put forward to members of the AB to understand the order of events and clarify the flow of processes within each phase of the pathway.

• Step 3: Advisory board consensus meeting

An online AB meeting was conducted in February 2023 to obtain consensus on key factors and potential barriers related to SDD after Micra™ implantation that emerged from the individual interviews. Consensus on discussion points was determined using an online survey (Table 1). Consensus was reached when at least 4 out of 6 AB members agreed with or were neutral on a specific discussion point. Furthermore, the proposed SDD after Micra™ Implantation Patient Pathway was also evaluated and changes to the pathway were proposed and agreed upon. 5 AB members attended the meeting; the last AB member provided their answers to the survey with comments about the pathway via email after the consensus meeting.

• Step 4: Finalisation of the SDD after Micra™ implantation pathway

**Table 1:** AB meeting survey results.

**Phase 1: Pre procedure assessment**		
**Question**	Response by AB*	**Comments by AB**
Do you agree that patients with comorbidities such as cancer and anticoagulation disorders should not be discharged on the same day?		One AB member stated “comorbidities such as cancer and anticoagulation disorders” is not totally correct, since there is a broad spectrum of patients with cancer and for those who are stable, SDD is feasible. He proposed to stipulate “patients on active anticancer treatment”. Another AB member stated that cancer and anticoagulation disorders should not be put together since they are two separate things. He also stated that an anticoagulation disorder would preclude SDD, while that’s not necessarily the case for a cancer patient. Others proposed that the only suggestion should be “significant or advanced comorbidities would preclude SDD”, but the pathway shouldn’t be as prescriptive – the physician and the team looking after the patient should make that decision.
Do you agree that patients with a very high/very low BMI should not be discharged on the same day?		One AB member stated that very high BMI and very low BMI are two conflicting issues and should be considered separately. Another AB member stated that BMI doesn’t make a difference one way or another. Members agreed that “very low/high BMI” should not be included in the pathway since they only have impact if related to bleeding issues or to other comorbidities.
Do you agree that age is not a cut‐off for SDD if we are assessing frailty, BMI and comorbidities?		No comments were raised during the meeting; all AB members agreed that age is not a cut-off for SDD.
Do you agree that PM dependency is not relevant for SDD choice?		None

**Phase 2: Micra™ implant**		
Do you agree that SDD should be avoided in case of issues with the recovery from sedation?		None
Do you agree that the type of vascular closure is key for SDD?		One AB member stated that the choice should be what the operator is familiar with.
What kind of vascular closure method should be adopted to increase chances for SDD? (multiple choice question)	Vascular Closure Device Figure of eight suture	None

**Phase 3: Post procedure observation and discharge**		
Do you agree that complications such as pericardium perforation, pericardial effusion, very severe vascular complications, severe hematoma, bleeding from access site and device complications prevent SDD?		None
Rest in bed flat or sit up at 30‐45° (from 2 to 4 h after procedure) and suture cut and mobilization (from 4 to 6 h after procedure): do you agree that these time points are accurate?		None
Do you agree that chest X‐ray, device check, electrocardiogram, access site evaluation is an accurate list of pre‐discharge tests/assessments?		It was established that there were differences amongst the AB members’ standard of care for tests/assessments before discharge. After discussing, AB members agreed that a difference should be established in the degree of recommendation for the different tests and assessments within the pathway.

**Phase 4: Follow-up**		
First in person follow‐up from 1 to 3 weeks after procedure (Micra™ AV) and 2 to 4 weeks after procedure (Micra™ VR): do you agree that the time points for follow up of Micra™ AV/VR are correct?		Most AB members disagree that there should be a difference in time‐point of first follow‐up visit between Micra™ VR and Micra™ AV patients.
Do you agree that long term follow up after SDD should be as per Micra™ standard of care of each centre?		None
Do you agree that remote monitoring could replace in‐person monitoring after the first visit?		One of the AB members said he was not comfortable recommending remote follow-ups as standard of care. It could be considered as an option, but not as a standard. Another AB member agreed with him regarding the first visit, but thereafter he didn’t find any major benefits in seeing the patient face to face if adequate remote monitoring is available, and if it is also the preference of the patient.

Agree Neutral Disagree.

* Each box under the response by AB represents the view of a member of the AB.

Suggestions from all AB members for improvement of SDD flow were considered and incorporated into the final version of the SDD after Micra™ implantation pathway presented in this consensus document.

## 3 Results

### 3.1 Benefits and limitations of the SDD strategy

The benefits and limitations of the SDD strategy were explored during individual interviews. Most experts agreed that the SDD strategy prioritised patient preference, as most patients would prefer to go home as soon as possible. From the implanting centre’s perspective, the SDD strategy may also provide a financial advantage and increased bed availability. Regarding limitations, the AB expressed concern about patient safety due to the lack of monitoring and inability to immediately attend to patients in case of complications after the procedure. The experts also foresaw logistic challenges in the scheduling of procedures, as patients should be monitored for 6 h after the procedure prior to discharge. This strategy was not perceived applicable for in-patients receiving Micra™ implantation. Additionally, SDD was ranked as a non-critical priority by the AB members (the general rating to the question “How much of a priority is SDD for you?” was 3 to 4, where 5 was very much a priority and 1 not a priority at all), and the main concern was patient safety. Therefore, effort was focused on optimising patient safety at all stages of the SDD patient pathway.

### 3.2 Patient pathway

**Fig. 1:**
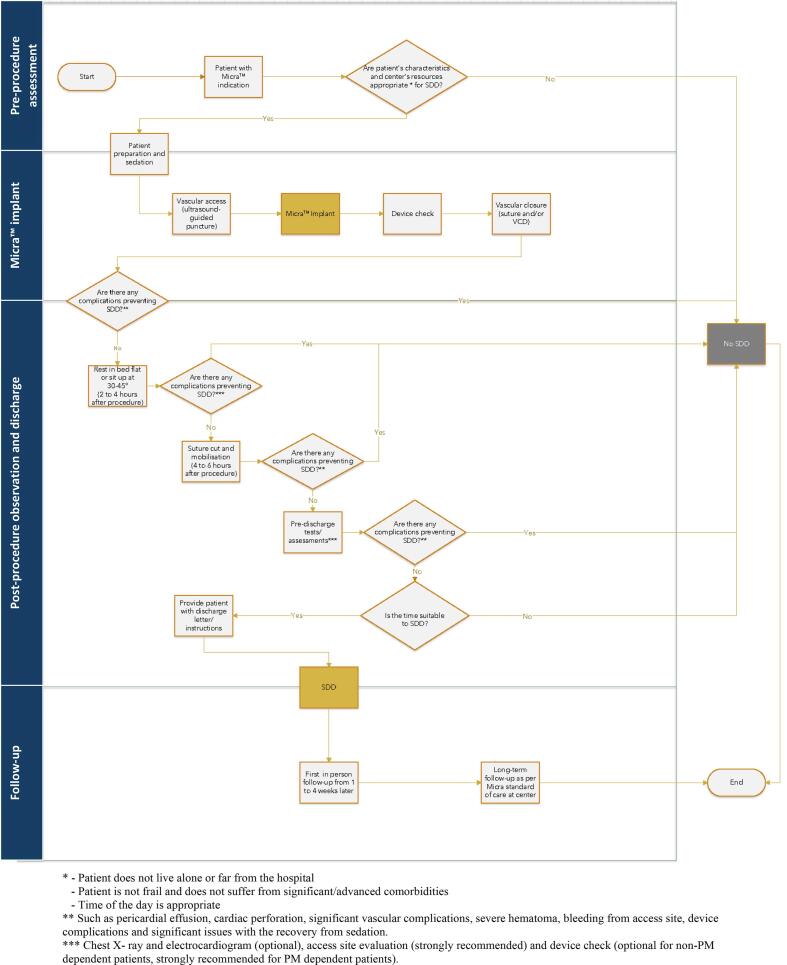
Same Day Discharge after Micra™ Implantation Pathway.

The SDD after Micra™ Implantation Patient Pathway is shown in Fig. 1. The objective of the pathway is to provide guidance to medical teams on how to safely implement SDD after Micra™ implantation. The pathway consists of four phases: 1) pre-procedure assessment; 2) Micra™ implant; 3) post-procedure observation and discharge and 4) follow-up. The key factors to consider before and after Micra™ implantation when determining the suitability for SDD are summarised in the Graphical abstract.

### 3.3 Phase 1: Pre-procedure assessment

The first phase of the pathway describes patient and centre-related factors that should be considered prior to the procedure to decide whether SDD is appropriate. The AB suggested that the use of a standardised checklist might be useful in facilitating thorough assessment of relevant factors. Table 2 shows an example of a checklist summarising key factors to be considered pre- and post- procedure to ensure safe SDD after Micra™ implantation.

**Table 2:** Checklist for consideration of Same Day Discharge after Micra™ implantation.

#### 3.3.1 Patient clinical factors

The first clinical factor to consider is the patient’s comorbidity burden. The AB concurred that the presence of **significant or advanced comorbidities, including frailty,** is an important indicator against SDD (Table 1). The severity and burden of comorbidities should be evaluated on a case-by-case basis by the treating medical team / operator. The specific list of comorbidities to consider in the pre procedure checklist would be for the treating physician to assess (Table 1).

In relation to the patient’s baseline characteristics, the effect of body mass index (BMI), age, pacemaker dependency and anticoagulation use on SDD were explored during individual interviews and the consensus meeting. Most AB members did not agree with (4/6, 66.67%) or were neutral to (1/6, 16.67%) the statement that “patients with very high or very low **BMIs** should not be discharged on the same day after Micra™ implantation”. The AB agreed that there should not be any **age** cut‐off for SDD if other clinical factors e.g. comorbidities, frailty status and BMI were evaluated (6/6, 100%). All AB members agreed that **pacemaker dependency** should not impact SDD (6/6, 100%). A similar result was observed for **routine anticoagulation** during the individual interviews (Table 1).

The **type of Micra™** (AV or VR) was considered not to influence the decision for SDD during individual interviews. The AB agreed that no **specific tests/assessments** were required for potential SDD patients on the day prior to implant.

#### 3.3.2 Patient social factors

All AB members pointed out during individual interviews that **social factors** were key in deciding if a patient could be discharged on the same day after Micra™ implantation. SDD may not be appropriate in patients with inadequate social support, for example, patients who live alone or who do not have a suitable caregiver or family to support them after discharge; patients with no fixed abode or insanitary living conditions; patients with no access to a telephone for emergency services; patients with no suitable means of transport or who require long distance (> 1 h) travelling to and from the hospital. It is particularly important to consider patient preference when making decisions about SDD. This allows the medical team to understand the patient’s agenda and explore their concerns, ensuring delivery of patient-centred care. Ideally, the patient and their carers or family should be informed about the possibility of SDD when the procedure is scheduled or at the pre-assessment visit, so that necessary arrangements could be made in advance to facilitate SDD.

#### 3.3.3 Centre-related factors

Consensus from the AB was that the **type of centre** (tertiary and/or high-volume centres versus small centres) could facilitate the Micra™ implants (or not, in case of small centres that do not have the necessary resources), but this should not impact the feasibility for SDD.

Regarding the location of the hospital, AB members specified that patients living in rural areas tended to stay in hospital overnight because of concern relating to delayed medical attention in the event of complications. This could be the case for some centres in Australia or in the Unites States of America, but not so common for centres in France, Spain, or the United Kingdom, where distances to centres are considerably shorter.

Regardless of the type of centre or its location, all AB members agreed that it is preferable to **schedule the procedure early in the day,** to allow a sufficient period of post-procedural monitoring, resumption of baseline ambulatory status and SDD at a reasonable time. The day of the week, however, was not identified as a general concern for SDD, as this was rather centre-specific. The medical team/operator should also ensure adequate staff and resources are available to efficiently manage any post-procedural complications following SDD.

### 3.4 Phase 2: Micra™ implant

The second phase of the pathway highlights important considerations for SDD during the procedure itself.

#### 3.4.1 Patient preparation

“Patient preparation” is placed halfway between Phase 1 and Phase 2 in the pathway to highlight that the process of patient preparation generally begins outside the catheterization laboratory. All experts considered **standard patient preparation** adequate for patients who will be potentially discharged on the same day of the procedure.

#### 3.4.2 Sedation

Regarding sedation, AB members agreed that **sedation protocols should not differ** between SDD and overnight patients. Additionally, general anaesthesia, though rare during Micra ™ implants, should not be an impediment for SDD. However, AB members agreed during the meeting that SDD should be avoided in patients who encountered significant issues during recovery from sedation (5/6, 83.33%).

#### 3.4.3 Vascular access, device check & closure

Regarding vascular access, all AB members agreed that **ultrasound-guided vascular access** is associated with fewer complications and therefore maximises the likelihood for SDD. After Micra™ implant, all patients (SDD or not) should have a device check before vascular closure. No differences have been identified in closure methods in SDD patients compared to those staying in hospital overnight. During the meeting, three AB members did not agree that the type of vascular closure is key for SDD (50.0%), and two were neutral (33.33%) regarding this point. The board agreed that vascular closure device (5/6, 83.33%) and figure of eight suture (4/6, 66.67%) should be adopted to increase the likelihood for SDD, although no specific closure method was recommended by the AB (Table 1).

### 3.5 Phase 3: Post-procedure observation and discharge

The third phase of the pathway details all steps that should be followed during observation period post-procedure, from when the patient leaves the catheterization laboratory until discharge.

#### 3.5.1 Peri-procedural complications

Multiple decision points regarding the evaluation of ***peri*-procedural complications** have been inserted throughout this phase to emphasize the importance of being vigilant about potential complications throughout the observation period. The first complication-related decision point is placed between phase 2 and 3 to highlight monitoring for complications should commence during the intraoperative and early post-operative period, prior to the patient returning to the ward. All members of the AB agreed that complications such as pericardial perforation, pericardial effusion, severe vascular complications, severe hematoma, bleeding from access site and device complications would prevent SDD (6/6, 100%). Other complications such as significant issues during recovery from sedation or complications unrelated to the procedure, could also preclude SDD. In order to capture potential complications, AB members concurred that the **minimum observation time** before discharge should be six hours.

#### 3.5.2 Post-procedure observation

All members of the AB agreed that patients should rest in bed flat or sit up at 30-45° for at least 2 to 4 h (6/6, 100%) post procedure. If the medical team/operator does not identify any complications, the SDD pathway continues. The next steps are suture cut and mobilisation, which the AB has confirmed should happen from 4 to 6 h post procedure (6/6, 100%). Similarly, the presence of complications is evaluated after this step, and if none are found, SDD should be considered for the patient.

#### 3.5.3 Pre-discharge tests and assessments

During the interviews, all AB members confirmed that tests and assessments are always carried out in Micra™ implanted patients prior to discharge. Potential pre-discharge tests and assessments include chest X‐ray, electrocardiogram (ECG), device check and access site evaluation. During the meeting, it became apparent that the standard of care tests and assessments prior to discharge vary greatly amongst centres. Therefore, the AB did not make any specific recommendations in this regard within the pathway. **Chest X‐ray, ECG and device check** are optional pre-discharge tests and should be performed according to the local policies at the implanting centre. Furthermore, the AB strongly recommended **access site evaluation** prior to discharge. Performance of a device check is also highly encouraged for pacing-dependent patients, as their well-being is dependent on optimal device performance. If no complications are identified after the above pre-discharge tests and assessments, the patient could proceed to SDD.

AB members concurred during the meeting that the feasibility of SDD should be re-confirmed, considering the timing of discharge, once all observations and pre-discharge assessments have been fulfilled. Additionally, all members stated that patients and their caregivers should be provided with instructions on symptoms to look out for, actions in case of groin hematoma or bleeding, and relevant emergency numbers and contact information before discharge.

### 3.6 Phase 4: Follow-up

The final phase of the pathway describes the recommended steps for the follow-up of patients after SDD.

#### 3.6.1 Short-term follow-up

The AB recommended that the first follow-up post SDD should take place via an in-person approach, between week 1 to 4 post procedure for both Micra™ variants. Most AB members do not think that the time-point of first follow-up should be different between Micra™ VR and Micra™ AV patients (4/6, 66.67%). However, earlier follow-up could be considered for Micra™ AV patients to confirm that AV synchrony is working as expected.

#### 3.6.2 Long-term follow-up

All members of the AB agreed that long-term follow up after SDD should be as per Micra™ standard of care at each centre (6/6, 100%). The effects of remote monitoring on SDD were discussed at the meeting and the majority of the AB agreed that remote monitoring could replace in‐person monitoring after the first visit (4/6, 66.67%).

## 4 Discussion

LP implantation has evolved in efficacy and safety, such that SDD could be considered in many patients. SDD has been demonstrated to be feasible and safe in the majority of patients referred for CIED. [10] In addition, SDD is often preferred by patients and may reduce procedure-related costs, increase bed capacity and savings for healthcare systems. [5] In a recent study of 81 patients who underwent elective Micra LP implantation, 44 patients were safely discharged on the same day after the procedure. [9] Compared to patients who were observed overnight, those who had SDD were younger (mean age 49 vs 67 years); the authors therefore suggest that SDD potentially could be applicable to young patients who are physically active and have low comorbidity burden. [9] There is currently no consensus on how to select patients for safe SDD and what the optimal timing of discharge for these patients is. This document provides practical guidance around SDD after Micra™ implantation and presents a patient pathway supplemented with pre- and post-procedure checklists to facilitate the safe implementation of SDD after Micra™ implantation.

The implementation of such an SDD pathway might require significant changes in workflow and may not be straightforward. Identification of appropriate staff to fulfil various roles within the pathway is key to its successful delivery. In addition to the outpatient cardiology clinical staff and catheterisation laboratory operational staff, other members such as ward staff in the pre- and post-procedure areas, administrative staff as well as pharmacists, all play an important role in facilitating smooth implementation of the pathway. Tasks such as completing the pre- and post-procedure checklists, communicating, and discussing the possibility of SDD with patients and their family, monitoring patients post procedure for complications and ensuring completion of relevant pre-discharge assessments and patient education, all require significant amount of staff time and buy-in. While SDD can save resources, the implementation might lead to a higher temporary workload.

Local champions may be particularly useful in helping to kick start and drive changes required to implement the SDD pathway. Physician champions are encouraged to work with their local team to develop a site-specific protocol for SDD and make use of the pre- and post-procedure checklist presented in this document. Furthermore, it is important to arrange meetings with relevant staff (advanced practitioners, nurses, pharmacist, administrative staff) to discuss their specific roles, so to reduce barriers to implementation and provide a forum in which staff could offer input into tailoring the pathway according to the needs of the patient population and healthcare facility.

Successful implementation of the SDD patient pathway has the potential to create better healthcare and clinical outcomes for patients with Micra™ implantation. First, the pathway addresses various patient and healthcare facility related factors to ensure safe patient recovery, education and follow up. Second, shared decision making is widely acknowledged throughout the pathway, optimising the delivery of patient-centred care. Third, the SDD checklist could potentially be integrated into the patient’s health record, which could simplify and shorten the discharge process. For example, certain elements of the checklist, including instructions about medication, follow up care, education about symptoms to look out for in case of complications, could be incorporated into the discharge letters and given to patients. Finally, adoption of the SDD pathway could potentially widen the patient population eligible for safe SDD in the future. For example, if SDD is successfully implemented in patients admitted for elective Micra™ implantation, this pathway could potentially be extended to other populations, such as those receiving Micra™ implantation as an in-patient. This could lead to greater patient satisfaction as well as increased savings within the healthcare systems.

Our study has limitations. First, this pathway is based on the consensus opinion of six high-volume Micra™ implanters, which, while ensuring a breadth of experience, may not fully capture the diversity of clinical practices and patient populations globally. Although there is a reasonable geographic variation among the implanters, the relatively small sample size may limit the generalizability of the findings. Second, the focus of this work is to develop a high-level pathway as a first step to serve as a potential SDD guide for clinicians located in different geographies. We did not study the clinical utility of the pathway on patients in a real-world setting; specific local practices, regulatory requirements or patient demographics could affect the pathway’s applicability in different regions and should be explored in detail in subsequent work. Additionally, the survey questions used to gather expert opinions aggregated multiple elements together. This approach may have led to some nuances being overlooked, and the responses might not fully reflect the complexity of individual clinical scenarios. Further studies are required to validate the SDD pathway described in the manuscript before wider implementation in different healthcare settings.

## 5 Conclusion

This consensus document outlines the development of a safe SDD after Micra™ Implantation Patient Pathway based on expert consensus of 6 experienced Micra™ implanters from across the world. The implementation of this pathway and related recommendations may facilitate safe SDD in patients electively admitted for Micra™ Implantation and has the potential to improve patient care and outcome and at the same time, reduce financial burden on healthcare systems.

## CRediT authorship contribution statement

**Riyaz Somani:** Writing – review & editing, Writing – original draft, Conceptualization. **James Daniels:** Writing – review & editing, Formal analysis, Data curation, Conceptualization. **Alexis Mechulan:** Writing – review & editing, Methodology, Formal analysis, Data curation, Conceptualization. **Vincent Paul:** Writing – review & editing, Methodology, Data curation, Conceptualization. **David Sharman:** Writing – original draft, Methodology, Data curation, Conceptualization. **Shirley Sze:** Writing – original draft, Methodology, Data curation. **Xavier Viñolas Prat:** Writing – review & editing, Methodology, Formal analysis, Conceptualization.

## Funding

This research did not receive any specific grant from funding agencies in the public, commercial, or not-for-profit sectors.

## Declaration of competing interest

The authors declare the following financial interests/personal relationships which may be considered as potential competing interests: Medtronic provided logistical support to the Advisory Board and covered the cost of the Article Processsing Charge.

## Data Availability

Data will be made available on request.
